# Segmentation of 3D images of plant tissues at multiple scales using the level set method

**DOI:** 10.1186/s13007-017-0264-5

**Published:** 2017-12-21

**Authors:** Annamária Kiss, Typhaine Moreau, Vincent Mirabet, Cerasela Iliana Calugaru, Arezki Boudaoud, Pradeep Das

**Affiliations:** 1 Laboratoire Reproduction et Développement des Plantes, Univ Lyon, UCB Lyon 1, ENS de Lyon, CNRS, INRA, 46, allée d’Italie, 69342 Lyon, France; 2 Centre Blaise Pascal, ENS de Lyon, 46, allée d’Italie, 69342 Lyon, France

**Keywords:** Confocal image, 3D, Segmentation, Level set method, Watershed, L1, Cell, Cellwall, Nucleus

## Abstract

**Background:**

Developmental biology has made great strides in recent years towards the quantification of cellular properties during development. This requires tissues to be imaged and segmented to generate computerised versions that can be easily analysed. In this context, one of the principal technical challenges remains the faithful detection of cellular contours, principally due to variations in image intensity throughout the tissue. Watershed segmentation methods are especially vulnerable to these variations, generating multiple errors due notably to the incorrect detection of the outer surface of the tissue.

**Results:**

We use the level set method (LSM) to improve the accuracy of the watershed segmentation in different ways. First, we detect the outer surface of the tissue, reducing the impact of low and variable contrast at the surface during imaging. Second, we demonstrate a new edge function for a level set, based on second order derivatives of the image, to segment individual cells. Finally, we also show that the LSM can be used to segment nuclei within the tissue.

**Conclusion:**

The watershed segmentation of the outer cell layer is demonstrably improved when coupled with the LSM-based surface detection step. The tool can also be used to improve watershed segmentation at cell-scale, as well as to segment nuclei within a tissue. The improved segmentation increases the quality of analysis, and the surface detected by our algorithm may be used to calculate local curvature or adapted for other uses, such as mathematical simulations.

**Electronic supplementary material:**

The online version of this article (10.1186/s13007-017-0264-5) contains supplementary material, which is available to authorized users.

## Background

Delineating the various processes underlying morphogenesis has been a key focus of developmental biology for many years. The quantitative assessment of shape changes in growing tissues and their correlation to genetic activities requires the accurate identification of its geometry at different scales, via both high-quality images from optical microscopy and the conversion of that information to a digitised format, a process known as segmentation.

In recent years, more and more image segmentation methods have been developed or improved upon (see [1] and references therein), open-source image analysis tools have become widely used in the biology community (see e.g., [2–4] and references therein). While the majority of these tools address the analysis of 2D images of biological tissues, in recent years 3D image analysis tools have also improved considerably both for plant [5–7] and animal species [8]. However, the accuracy of these tools is reliant upon the correct detection of tissue and/or cell contours.

**Fig. 1:**
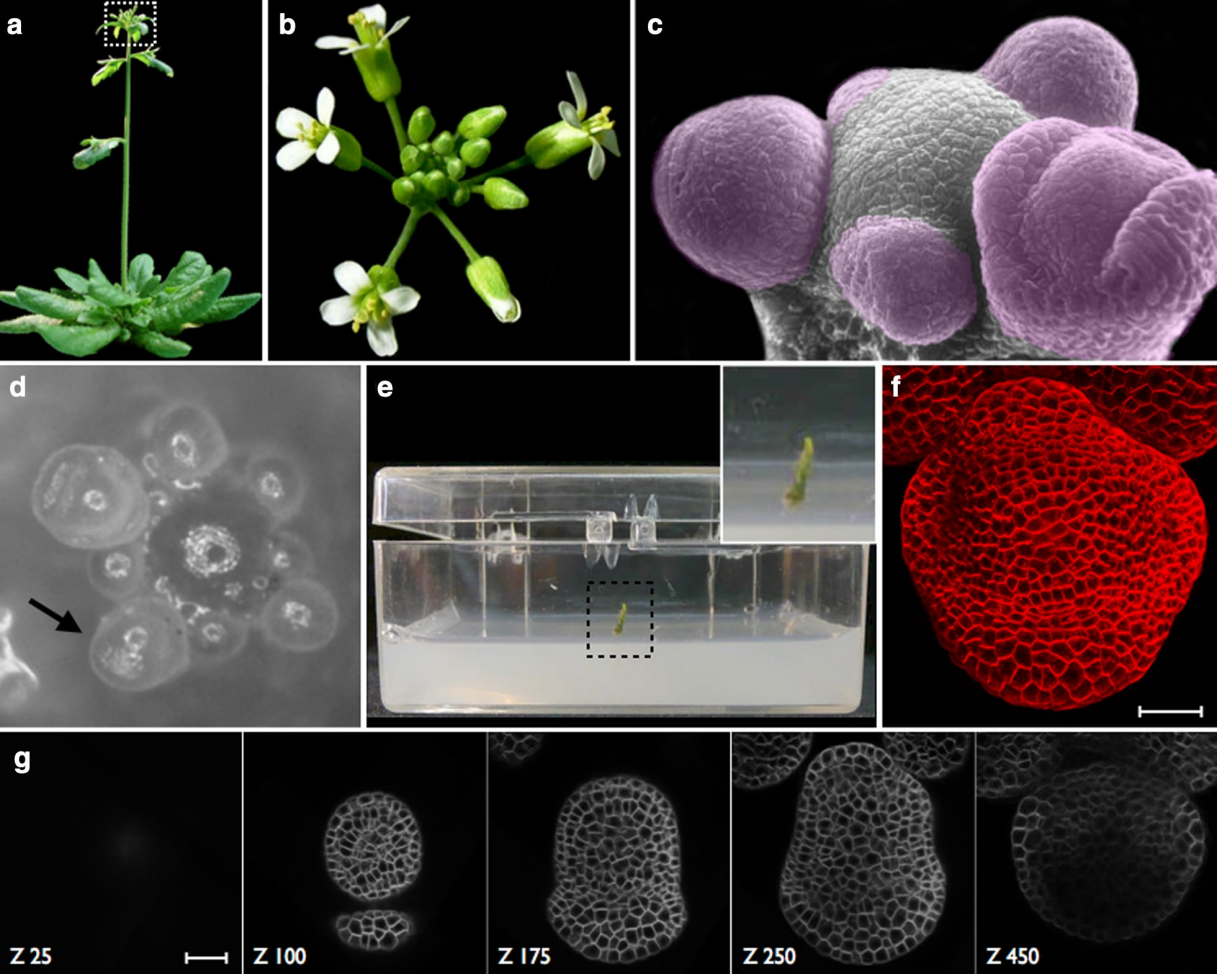
Description of the experimental context. **a** A side view of the aerial parts of an *Arabidopsis thaliana* plant. The boxed region indicates the position of the Inflorescence Meristem (IM) at the apical tip of the plant. **b** A top view of the IM, with mature flowers as well as young floral meristems visible. **c** A scanning electron micrograph of the IM with most flowers dissected away. Remaining flower buds are false-colored in violet. **d** A brightfield image of the same structure with a stage 3 flower bud indicated (arrow). **e** The dissected stem is stabilized on growth medium in a box suited to the water-dipping objective on the confocal microscope. **f** A 2D surface projection of the 3D image stack acquired on the confocal. **g** Individual slices from various positions along the Z axis indicate the 3D nature of the image stack. Z positions are provided. Scale bars indicate 25 μm

**Fig. 2:**
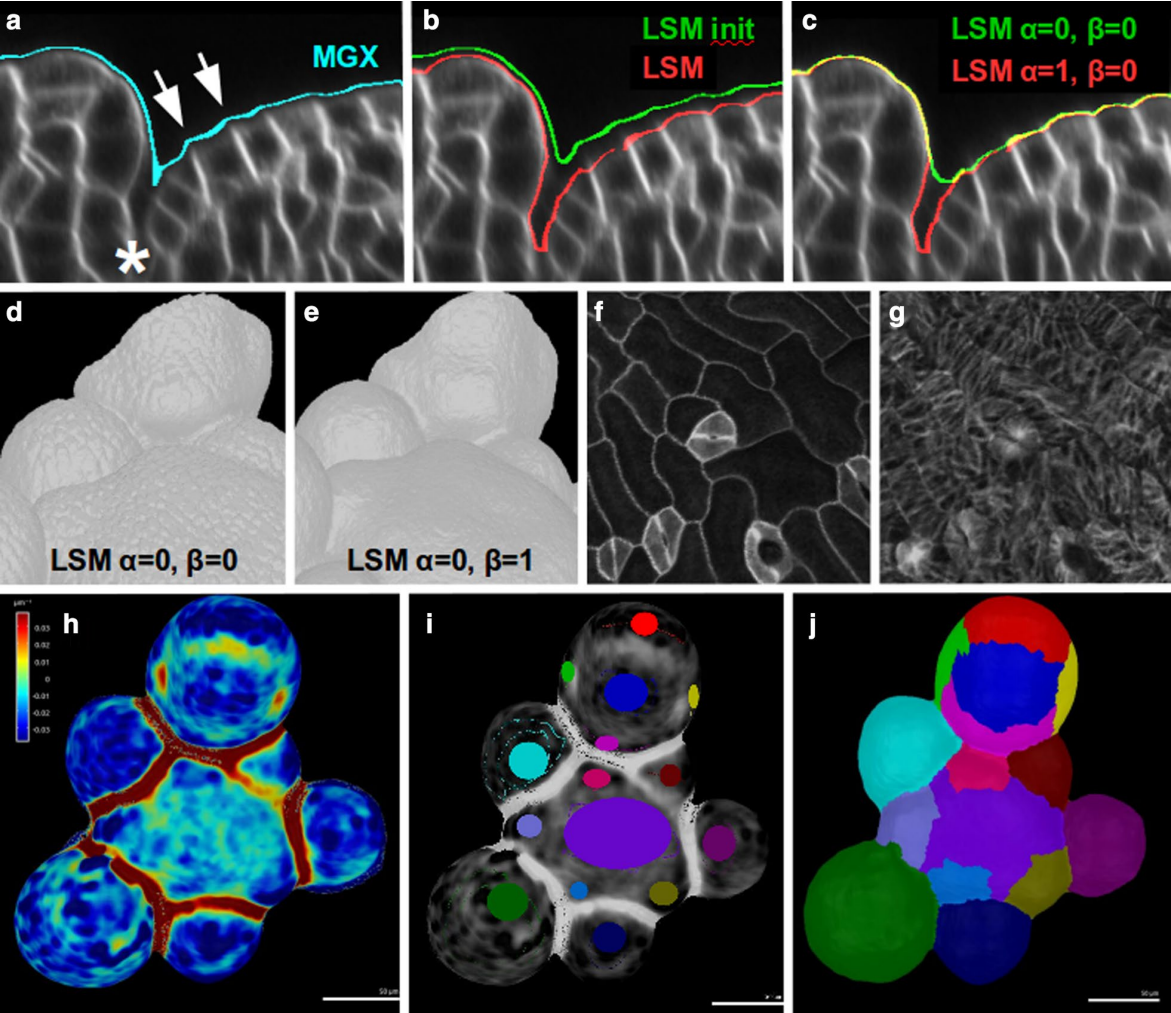
LSM for tissue contour. **a**–**c** Section of a developing flower at the boundary between the peripheral zone and the forming sepal. **a** Tissue contour detected with the “Edge Detect” process of the MorphoGraphX software. The contour doesn’t enter in deep creases (asterisk) and presents crests (instead of valleys) above the anticlinal walls (arrows). **b** Tissue contour detected with the level set method. The green contour is an initialisation, while the red contour is the result of the level set contour detection ($\alpha =1$, $\beta =0$). **c** The effect of the accelerating term weighted by the parameter $\alpha$ is to “push” the contour into profound valleys on the surface: green contour $\alpha =0$, red contour $\alpha =1$ and all other parameters are the same and default values. **d**, **e** The effect of the smoothing term weighted by the parameter $\beta$: **d** $\beta =0$ and **e** $\beta =1$ and all other parameters are the same and default values. **f**, **g** Accurate surface detection allows accurate signal projection on the surface. Here is an example of membrane signal (**f**) and microtubule signal (**g**) projected using MorphoGraphX on a surface detected with the level set method based on the membrane signal. **h**–**j** The surface detected with the level set method allows to do accurate curvature maps, which can be segmented in order to determine organ boundaries. Here we present the curvature map and its segmentation done with MorphoGraphX: **h** Average curvature map (on a neighbourhood of 10 μm). **i** Manual seeding of the curvature map. **j** Watershed segmentation of the map

**Fig. 3:**
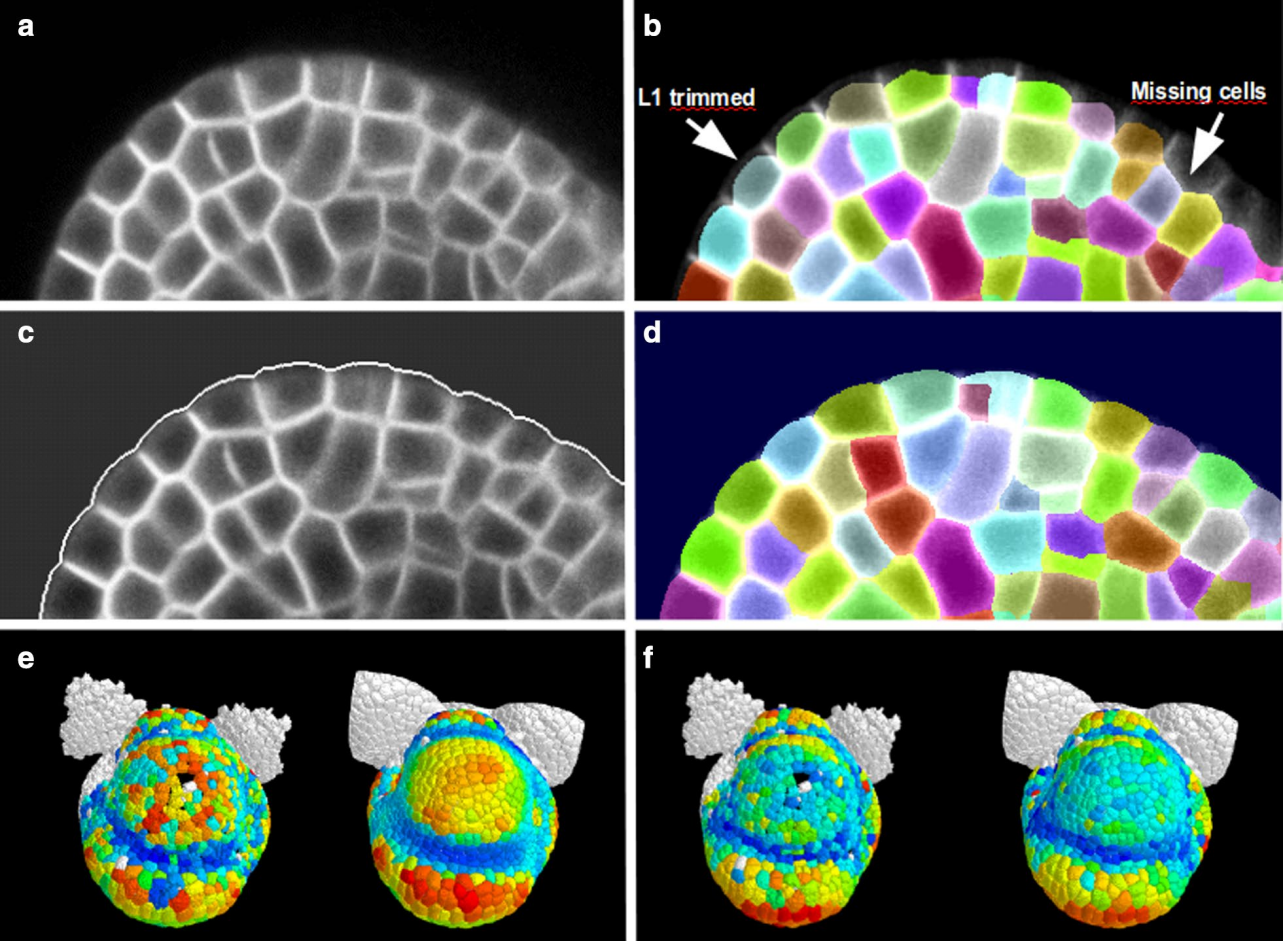
Cellular segmentation of a modified image with reinforced tissue contour. **a** Section of original 3D image to segment, where the outer periclinal walls are poorly marked. **b** The result of watershed segmentation (using MARS pipeline): the outer cell layer is trimmed and there may be even some missing cells. **c** Using the outer contour detected by the level set method, the outer periclinal walls are enhanced on the image, and the outer background is put to the most frequent value of the inner tissue. **d** The result of the watershed segmentation of the modified image. **e** Gaussian curvature and **f** cell volume is computed and represented first on data obtained by watershed segmentation of the original image, and second on data obtained with the improved MARS pipeline where the outer periclinal walls were enhanced using the contour level set before doing watershed

Tissue surface detection is particularly challenging in regions presenting creases or folds, such as at the boundaries between developing organs. One such example in plants is in developing flowers (Fig. 1a–f), where the region between the undifferentiated central dome and the growing sepal can form a deep fold (Fig. 1c, f as well as asterisk in Fig. 2a). Such regions present a serious challenge for the surface detection methods currently used in the community, where the use of a simple threshold in pixel intensity can only provide an approximate surface [7]. The identification of cell contours in images where the plasma membrane or cell-wall is marked (Fig. 1f, g) is frequently based on the watershed method [5, 6]. However, at least in the case of plant tissues, watershed segmentation methods routinely encounter difficulties with outer (periclinal) cell surfaces, which are more poorly marked than anticlinal surfaces (Fig. 3a). This is possibly because these outer cell surfaces contain only one membrane, whereas all inner surfaces contain two, making them easier to stain and image. Additionally time-course images are generally of sub-optimal quality since exposure times and laser intensities have to be minimised to avoid phototoxicity and related artefacts.

The watershed method identifies cell contours by seeking crests of high intensity that separate two low-intensity regions within an image. To overcome the issues discussed above, we complement the watershed step by using the level set method, which identifies outlines by seeking high-gradient regions that separate a single low-intensity region (such as background) from a higher-intensity region (such as the tissue of interest). Such gradient-based approaches have also been used for cell segmentation and tracking in zebra-fish embryogenesis [8–13]. Certain level set based methods have also been implemented in common image processing packages. For instance, the image analysis platform ImageJ [2, 14] has level set plugins, but for the moment only for 2D images, while the use of the level set implementations of the ITK library [15] require some experience in both image processing and programming.

Here we present a simple level set implementation for the segmentation of plant tissues at both organ- and cell-scale. We present the principles of the level set method as well as its parameters in “Methods” section. Then we demonstrate how this framework can be used to improve segmentation results in multiple contexts in “Results and discussion” section: detection of the tissue contour, segmentation of the outer cell layer, cellular segmentation within whole tissues, and segmentation of nuclei in whole tissues.

## Methods

### Plant growth and imaging

*Arabidopsis* plants were grown as previously described [16]. Briefly, an approximately 2 cm-long piece of the upper part of the stem was cut and placed in growth medium (Fig. 1a–e). The stem was then submerged in water and all flower buds older than stage 3 were dissected away. Immediately prior to imaging, inflorescences were treated with the water-soluble lipophilic dye FM 4-64 (Invitrogen), which labels cell membranes (Fig. 1f). The treated inflorescences were then observed on either a Zeiss LSM 700 or a Leica SP8 confocal microscope with 40× water immersion objectives. After the first image stack was acquired (Fig. 1g), the meristems were twice rotated and imaged again [5].

### Level set method

We first describe the general principles of our implementation of the level set method (LSM), which we then apply to images in several different contexts in the next section in order to detect the external contours of tissues or the contours of cells within 3D images. Mathematical definitions and implementation details are provided in the Additional file 1.

The approach is based on an evolving smooth contour, which is first suitably initialized (green contour in Fig. 2b) and then this contour evolves by seeking out and adhering to the outline of the sample (red contour in Fig. 2b).

This method is commonly used in image analysis to detect the outlines (contours or surfaces) of objects in a given image [17–20], as well as to easily trace their shapes regardless of changes in their topology [20, 21]. The contour is represented by the zero level of a higher dimensional function, called the level set function (LSF) and denoted by ${\phi }$. The “movement” of the contour is determined by the evolution of the LSF, so as to minimize a pre-defined energy functional, typically a linear combination of three energy terms and of a regularising term,

1 $$E_\varepsilon \ =\ \lambda \mathcal{L}_g\ + \beta \mathcal{L}_1 + \alpha \mathcal{A}_g + \mathcal{R}_p.$$

Given an initial LSF $\phi (x,0)={\phi }_0(x)$, this energy functional (1) can be minimized by solving the gradient flow

2 $$\begin{aligned} \frac{\partial {\phi }}{\partial t} = -\frac{\delta E_\varepsilon }{\delta {\phi }}. \end{aligned}$$

The most important term in this energy functional is the so-called *image term* $\mathcal{L}_g$, which guides the evolving contour to the desired structure on the image. It is based on a sum along the contour of an edge function *g*, which is generally related to image brightness and which is minimal on the structure we would like to detect.

The edge function *g*, appearing in the image term $\mathcal{L}_g$ is in general chosen to be of the form of a Hill function

3 $$\begin{aligned} g(\mathbf{{x}})=\frac{1}{1+\left| f(\mathbf{{x}})/\gamma \right| ^2}, \end{aligned}$$

with *f* taking maximal values on the target object and $\gamma$ a parameter. The choice of this indicator function *f* is one of the key points in adapting a level set method to a specific segmentation problem. We will see in the next section that different choices of this indicator function *f* [as in Eqs. (4–6)] lead to different results of the segmentation.

A special case of this term is when *g* is a constant, and since the sum is made along the contour, the shorter the contour, the lower this energy term is. Therefore, an $\mathcal{L}_1$ term in the final energy is considered apart, and can be used as an independent *smoothing term* for the contour.

The *accelerating term* $\mathcal{A}_g$ is also used in level set formulations, which is based on a weighted area (volume in 3D) of the domain enclosed by the contour. This term results in an acceleration of the movement of the contour when it is distant to the object boundaries and a deceleration when the contour approaches the target object.

Finally, we also added a *regularising term* $\mathcal{R}_p$, solely in order to create numerical stability (see the Additional file 1 for more details).

To summarize, level set method for image segmentation comprises the following steps:

1. *Initialization of the LSF within the 3D image*. A suitable initialization is a coarse estimation of the contour to detect. It can be provided as threshold (intensity) values that may differ at the top and bottom of the image. But it can also be provided as any other already existing coarser segmentation. The exact initialisation does not affect the final outcome (see Additional file 1: Figure 1E), but only affects computation time.
2. *Definition of the indicator function* *f* *and its scaling parameter* $\gamma$. The indicator function guides the LSF towards the target object within the image. Its scaling parameter $\gamma$ depends in particular on the span of *f*-values within the image, and as such on the quality of the image.
3. *Choice of parameters* $\alpha$ *and* $\beta$. The weight parameters $\alpha$ for the accelerating term and $\beta$ for the smoothing term are defined relative to the fixed $\lambda$ weight of the image term. In order for the contour to remain faithful to the image, the parameters $\alpha$ and $\beta$ must be kept low with respect to $\lambda$. The choice of values is left up to the user, and would depend on the specific biological context under investigation. A brief analysis of how these parameter values affect the contour is provided (Additional file 1: Figure 1).
4. *Run Equation* (2) *until convergence*. The stop criterion is based on the deceleration of the contour in the vicinity of the solution.

Given the above presented general framework, in what follows we apply these steps to several specific segmentation problems by specifying points 1, 2 and 3 of the LSM. The optimal choice of parameter values depends on the quality of the fluorophores and on the biological sample, while the convergence of the algorithm also depends on the shape of the object being studied [22]. While this may appear complex, it is important to note that in our hands, a fixed set of parameter values gives us stable results in different tissues and even organisms.

Our recommendation for choosing the right parameter values would be to begin by initialising LSM to stay as faithful as possible to the image ($\alpha =0$, $\beta =0$). After inspecting the results, the user might vary the accelerating and smoothing terms depending on the desired outcome.

## Results and discussion

The general LSM approach can be easily adapted to different contexts, such as to address specific problems of segmentation. Here we present LSM-based solutions for four such contexts.

### Level set for tissue contour

An accurate quantitative analysis of tissue growth requires the faithful detection of the outer surface of the tissue under consideration, in addition to cellular or subcellular features of interest. The task becomes challenging when the tissue begins to crease, for instance at a slow-growing boundary between two growing organs (Fig. 2a, b). In order to increase the reliability of surface detection in such areas, we have applied the level set method in the following manner.

We first initialize the level set at the exterior of the tissue using appropriate threshold values so that the contour is set close to, but not in contact with, the surface (green line in Fig. 2b). Images typically lose brightness when descending along the z axis (which corresponds to the axis of acquisition of the image stack). Therefore we implemented a threshold function that varies linearly along the z axis. Using ImageJ or an equivalent software, the user may manually determine the initialisation threshold parameter values at the desired distance from the sample at the top and bottom of the image, and may provide these threshold values as input parameters. Notice also that in general, the anticlinal walls are much brighter than periclinal outer walls, producing a brighter zone in the crease between cells. On this initial surface this will produce crests in these zones (see white arrows in Fig. 2a), showing that a surface detection based just on thresholding cannot be faithful to the shape of cells.

**Fig. 4:**
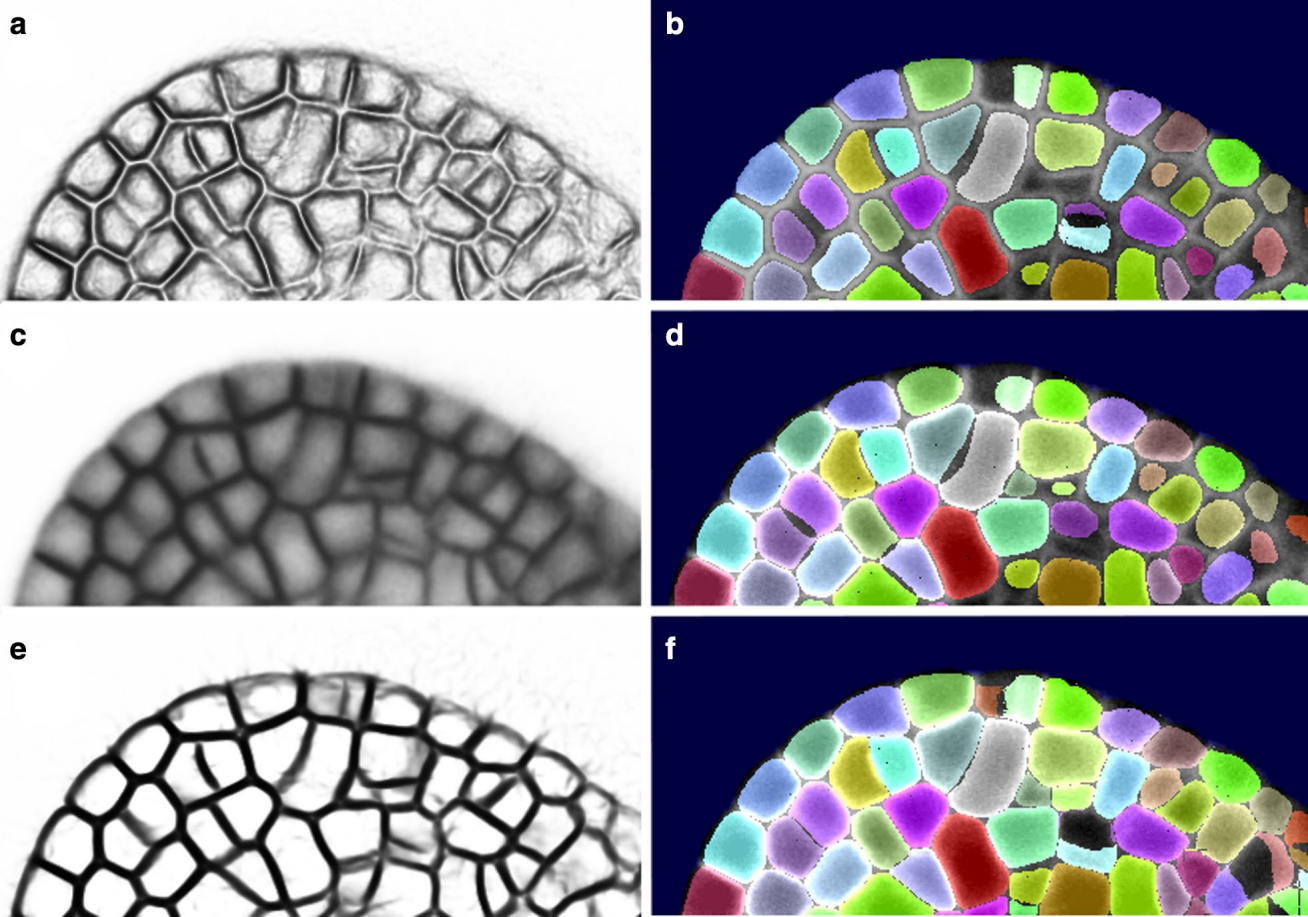
LSM for cellular segmentation. **a**, **c**, and **e** Show different indicator functions *g* and **b**, **d**, and **f** show the corresponding cellular segmentations. **a** The indicator function *g* based on the gradient $f_1$ with $\gamma =8$. **b** Gradient based cellular level set segmentation ($\alpha =1$, $\beta =0$, $\gamma =8$, $s=1,$ type = ‘g’). **c** The indicator function *g* based on the image intensity $f_2$ with $\gamma =60$. **d** Image based cellular level set segmentation ($\alpha =1$, $\beta =0$, $\gamma =60$, $s=1$, type = ‘i’). **e** The indicator function *g* based on second order derivatives of the image $f_3$ with $\gamma =0.8$. **f** Hessian based cellular level set segmentation ($\alpha =1$, $\beta =0$, $\gamma =0.8$, $s=1$, type = ‘h’)

Thereafter, a suitable indicator function is defined, that is a specific *f* in (3) is given. Here we use the most frequently used gradient based indicator function [19, 23], which guides the evolving contour towards the edge of the nearest object,

4 $$\begin{aligned} f_1(\mathbf{{x}})\ =\ |\nabla \hat{I}| \end{aligned}$$

where $\hat{I}$ is a smoothed version of the image *I*. For our typical image (Fig. 3a) this gradient-based indicator function *g* is represented in Fig. 4a. Once the LSF is initialised on the exterior of the sample (green line on Fig. 2b), it accelerates towards the tissue surface, slows down as it gets close to it, before finally stopping at the tissue surface (red line on Fig. 2b).

The principal parameters of the algorithm are the $\alpha$ and $\beta$ coefficients that describe the accelerating and smoothing terms, respectively. The same way as in the creases between cells on the surface of the tissue, there is a halo of bright pixels within the folds of tissue that separate individual organs or regions. If these folds are deep and if the image is bright, the contour might not be able to enter the crease in the absence of an accelerating term ($\alpha =0$). The accelerating term typically forces the contour into the crease (Fig. 2c, where we compare $\alpha =0$ in green and $\alpha =1$ in red and all other parameters are the same and of default values).

The smoothing parameter $\beta$ is of use when, instead of fidelity to all cellular details (Fig. 2d with $\beta =0$), one wishes to detect global tissular aspects (Fig. 2e with $\beta =1$). This is the case for tissular curvature maps, where accurate surface detection is important without cellular details on the surface [24].

An interesting observation is that mean curvature maps on 3D surfaces have maximal values in creases. Thus the map can be segmented using watershed segmentation on the surface and can be used to automatically identify the boundaries between different organs. In Fig. 2h of an *Arabidopsis* meristem with growing flower primordia we show the mean curvature map computed with the software MorphoGraphX [7] on the surface detected with the level set method. After a manual seeding shown in Fig. 2i, where each desired region was marked with different labels, a watershed segmentation is performed on the surface, and the boundaries between flower primordia and the central zone as well as the boundaries between sepals and the centre of the biggest flower could be detected automatically.

Another situation where using the level set method for an accurate surface detection can be crucial is for detecting and projecting signals from one or more different fluorescent channels at a defined distance from the surface of the sample. For example, cortical microtubule signal within a distance of 1 μm from the cell surface can be projected onto the detected tissue surface [25].

### Segmentation of the outer cell layer

The MARS pipeline [5] represented a large advance in robustly reconstructing and segmenting 3D confocal images of growing tissues at cellular resolution. However, because the outer walls are often less visible than the internal walls (Fig. 3a), the segmentation of the outer cell layer (called L1) is often error-prone (Fig. 3b). In particular, the watershed segmentation algorithm systematically results in incomplete cells at the surface of the sample, and others that are entirely missing. The trimming error is due to the fact that the background intensity is much lower than the mean intensity inside the cells. The missing cells in the first cell-layer are caused by the fact that the external cell membranes are much less marked than anticlinal and inner ones. So the use of global parameters that do not yield excessive segmentation in the inner tissue, does not permit to find seeds as local intensity minimum in these outer cells.

To overcome these problems, we detect the contour of the tissue using the level set method described above (“Level set for tissue contour” section). Then, we generate a modified image for the segmentation algorithm (Fig. 3c), in which the outer surface is artificially set to white, while the background intensity is chosen as the most frequent value inside the tissue (a coarse but simple way to estimate the mean intensity in the interior of the cells). The segmentation generated using this modified image does not display any trimmed cells on the surface, nor does it contain regions with missing cells (Fig. 3d).

In order to illustrate the improvement of the outer layer segmentation, we present a qualitative comparison of the Gaussian curvature of the surface (Fig. 3e) as well as the cell volumes (Fig. 3f) computed on a segmentation obtained by the watershed segmentation of the original image (left) and of the modified image (right).

### Cellular segmentation with level set

We next considered the question of whether the level set method could be used to improve the segmentation of individual cells within the tissue by smoothening cell contours. To carry out the cellular segmentation of the tissue, we initialize as many LSFs as the number of cells in the tissue and initialise them in the interiors of each cell. This initialisation could be either based on the seeds detected during watershed segmentation process (see for instance [5]) or, if a segmented image is available, on eroded segmented cells.

Depending on the indicator function and parameters one chooses for the evolution of the contour, different types of segmentations can be achieved, even if the parameters $\alpha$ and $\beta$ are kept constant: with $\alpha =1$ the accelerating term is “inflating” cells and it is pushing the cell-contour to the cell wall, while with $\beta =0$ the smoothing term is off and allows the contour to deform and to follow the guidance of the indicator function linked to the image.

The gradient-based indicator function *g* (Fig. 4a) with $f=f_1$ defined in Eq. (4) presents double valleys for each interior cell-wall (one valley for each side of the wall), keeping the detected cell-contours at a distance from each-other (Fig. 4b). Given this inner cell boundary segmentation as well as the outer background, the usual way of obtaining a segmentation of the entire tissue with no cell-wall thickness is to attribute each remaining voxel to the closest segmented object [8, 13].

Here, in order to obtain a complete cellular segmentation, we use an indicator function that presents only one valley for a cell-wall, so that the outline of neighbouring cells obtained using the level set method can come into contact. To do this we might employ the simplest approach of using the image intensity itself as function *f*, as in [26],

5 $$\begin{aligned} f_2(\mathbf{{x}})\ =\ \hat{I}. \end{aligned}$$

This solution (for the indicator function *g* see Fig. 4c) gives a cellular segmentation given in Fig. 4d.

However, as in the anisotropic diffusion filter that enhances planar structures in the image [6], we introduced a wall indicator function that relies on second order derivatives of the image. In particular, if $\lambda _1(\mathbf{{x}})> \lambda _2(\mathbf{{x}}) > \lambda _3(\mathbf{{x}})$ are the eigenvalues of the local Hessian matrix of the smoothed image $\hat{I}$, then we choose for the function *f* in the indicator function *g*

6 $$\begin{aligned} f_3(\mathbf{{x}})\ =\ min(\lambda _3(\mathbf{{x}}),0). \end{aligned}$$

This indicator function is new in the context of level set methods, has the advantage that it shows sharp valleys on inner walls as well as on outer walls of the tissue (Fig. 4e), and the resulting segmentation contains cells with smooth contours that occupy more of the image, leaving fewer inter-cellular spaces (Fig. 4f).

### Segmentation of nuclear images

**Fig. 5:**
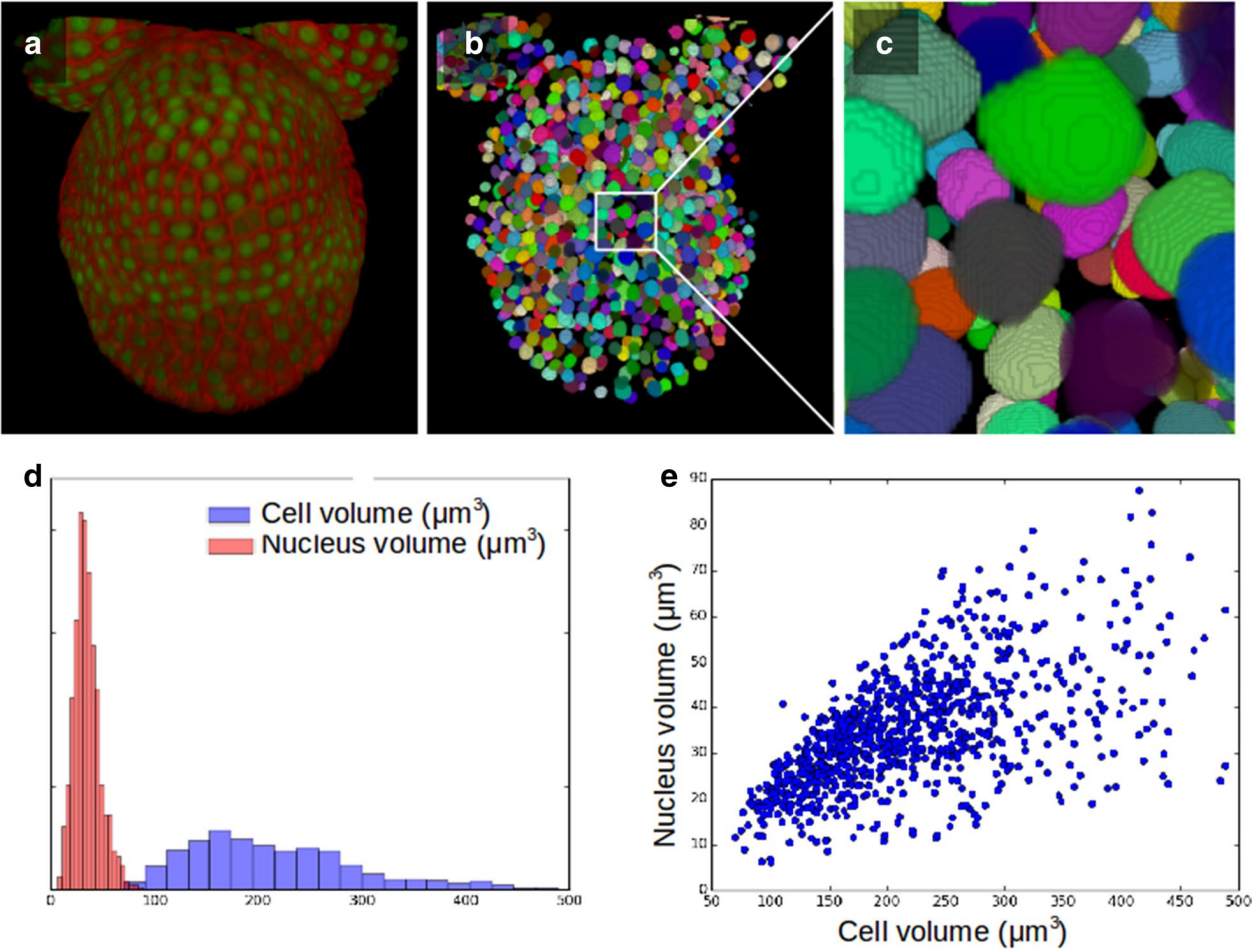
Cellular LSM used for the segmentation of nuclei. **a** Two-coloured confocal image of an *Arabidopsis* flower, were cell membrane and nuclei are taken on two distinct channels. **b** Segmentation of nuclei using level set method (erosion for initialisation $d=1$, other parameters $\alpha =0$, $\beta =0$, $\gamma =0.8$, $s=1$, type = ‘g’). **c** Zoom on the segmented nuclei. **d** Distribution of the volume of nuclei and cells in the tissue. **e** Nuclear volume versus cell volume, a correlation coefficient of 0.58 was measured

While segmenting tissues at cell resolution can be crucial to quantify growth, it can also be valuable to identify and characterise nuclei, in terms of their shapes and/or in terms of expression levels of a given reporter. To this end, the use of the cellular level set code can also be extended to segmenting nuclei in 3D images. We applied our code on high-resolution 3D reconstructed images obtained by the fusion of multiple confocal images taken at different angles, based on the MARS pipeline [5]. We use two-channel stacks, where a cell membrane marker and a nuclear-localised reporter were simultaneously imaged at different emission wavelengths (Fig. 5a).

To segment the nuclei using the level set method, we first initialise with an eroded cellular segmentation. The gradient-based indicator function can perfectly guide the evolving contour, while the parameters $\alpha =0$ and $\beta =0$ switch off the accelerating and smoothing terms and allow the contour to look for the shape of the nuclei within the initial contour (Fig. 5b, c). In our test image, a tissue of 1068 cells, nuclei were successfully detected in 88% of the cells. Where nuclei were not detected, an object of a couple of voxels was maintained in the segmented image, this can filtered out by its size, but keeps track of the existence of this cell in the initialisation.

The simultaneous segmentation of cells and nuclei allows in particular to measure the mean cell volume with its standard deviation ($217\pm 100$ μm$^3$)as well as the mean nucleus volume with its standard deviation ($33\pm 13$ μm$^3$). In vegetatively grown wild-type fission yeast *Schizosaccharomyces pombe* the ratio of nuclear to cell volume ratio was found to be $0.080\pm 0.013$, and a correlation coefficient between cell size and nuclear size of 0.68 was measured [27]. Here, in our test example of developing flower tissue, we find the ratio of nuclear to cell volume ratio $0.170\pm 0.067$ and a correlation coefficient of 0.58 between cell size and nuclear size. The distribution of cell- and nuclei volume are shown in Fig. 5d, while their correlation is illustrated in Fig. 5e.

## Concluding remarks

In the context of quantitative analysis of plant development, the accurate segmentation of images at both tissue- and cell-level is particularly relevant. Here we present a simple and efficient tool that can be easily used for detecting the contour of tissues, cells or nuclei.

The detection of tissue contours is particularly useful for studying the changing morphologies of biological systems during development. For example, the mean morphological development of leafs, as well as the variability around the mean behaviour was quantified using only their 2D contours at different developmental stages [28]. A key challenge in generalising such an approach to assess developmental trajectories for 3D tissues is the detection of accurate tissue contours, which our tool efficiently achieves.

Segmentation at the cellular level is useful to properly describe tissue structure, and to correlate genetic activity, via reporter expression, to cell specification and the regulation of growth through time. Our tool serves in two ways to increase the accuracy of standard watershed implementations in achieving cell-level segmentations. By ensuring that the outer surface of the tissue is faithfully detected, segmentation of the outermost cell layer is improved. This in turn prevents segmentation of the underlying cell layers from being influenced by errors at the tissue surface.

In plants, transcription factors play a key role as master regulators of diverse developmental transitions. These proteins tend to accumulate and be active in the nucleus, and as such, a quantitative analysis of their dynamics could be rendered more relevant by measuring their concentrations in nuclei, and by correlating this to factors such as nuclear size. Nuclear segmentations could also be useful in other contexts, such as in investigating nuclear volume changes at different phases of the cell cycle, or in measuring nuclear deformations in cells under mechanical compression.

### Availability

The contour-detection as well as the cellular level set code is written in C++ and uses the CImg image processing library [29]. The cellular level set tool is parallelized using OpenMP. They are put together in a package for Linux and Mac OS named *lsm3d*.

The standalone package *lsm3d* is distributed under a permissible licence for non commercial use, namely the Free Software licence CeCILL v2. The source code is accessible together with the associated installation and usage advises on the following webpage https://forge.cbp.ens-lyon.fr/redmine/projects/lsm3d. It was tested on Linux as well as on Mac OS systems.

## Additional file

**Additional file 1.** Supplementary Methods. **Section 1.** Mathematical details of the level set method; **Section 2.** Parameters and initialization; **Figure S1.** Parameter effects and sensitivity to initialization.
