# Supplementary material for: Segmentation of 3D images of plant tissues at multiple scales using the level set method
Source: Plant Methods. 2017 Dec 21;13:114. doi: 10.1186/s13007-017-0264-5 (PMC5738845; doi:10.1186/s13007-017-0264-5)
Supplement: Supplementary file 1 — Additional file 1. Supplementary Methods. Section 1. Mathematical details of the level set method; Section 2. Parameters and initialization; Figure S1. Parameter effects and sensitivity to initialization. [file 13007_2017_264_MOESM1_ESM.pdf]

# Segmentation of 3D images of plant tissues at multiple scales using the level set method

Kiss et al.

# Supplementary Methods

## 1 Mathematical details of the level set method

We have adopted the distance regularised level set evolution (DRLSE) [1]. In this formulation of the LSM a regularising term is included in the energy function in order to avoid the necessity of periodically reinitialising the LSF function. Simultaniously, despite the use of simple finite difference implementation and of relatively large time steps sufficient numerical accuracy is ensured by this formulation.

More precisely, let $\phi : \Omega \to \mathbb{R}$ be an LSF defined on a domain $\Omega$ having its zero level the evolving contour. Assuming that the embedding LSF function takes positive values inside the contour and negative values outside, the inward normal vector can be expressed as $\mathbf{n} = \nabla\phi/|\nabla\phi|$, while the scalar curvature is $\kappa = \nabla\mathbf{n}$.

The evolution of the LSF minimizes the energy functional

$$E_\varepsilon \;=\; \lambda\mathcal{L}_g \;+\beta\mathcal{L}_1 + \alpha\mathcal{A}_g + \mathcal{R}_p, \tag{1}$$

a linear combination of the following terms.

The *image term* $\mathcal{L}_g$ in this energy functional guides the evolving contour to the desired structure on the image,

$$\mathcal{L}_g(\phi) \;=\; \int_\Omega g(\mathbf{x})\delta(\phi)|\nabla\phi|\mathrm{d}\mathbf{x}, \tag{2}$$

where $\delta$ is the Dirac delta function, so that the energy $\mathcal{L}_g(\phi)$ is the integral of the edge function $g$

$$g(\mathbf{x}) = \frac{1}{1 + |f(\mathbf{x})/\gamma|^2}, \tag{3}$$

along the zero level contour of $\phi$.

The *accelerating term* $\mathcal{A}_g$ is based on a weighted area (volume in 3D) of the domain enclosed by the contour,

$$\mathcal{A}_g(\phi) \;=\; \int_\Omega g(\mathbf{x})H(-\phi)\mathrm{d}\mathbf{x}, \tag{4}$$

with $H$ the Heaviside function.

Lastly, we also added a simple *regularising term* with the potential $p(|\nabla\phi|) = \frac{1}{2}(|\nabla\phi| - 1)^2$, in order to prevent the numerical degradation of the LSF during evolution by maintaining its gradient close to unity without any reinitialisation following [1]

$$\mathcal{R}_p(\phi) \;=\; \mu\int_\Omega p(|\nabla\phi|)\mathrm{d}\mathbf{x}. \tag{5}$$

To summarize, the global energy functional in Equation (8) is the linear combination of all these terms, with the Dirac delta function $\delta$ and the Heaviside function $H$ appearing in $\mathcal{L}_g$ and $\mathcal{A}_g$ respectively approximated by the functions

$$\delta_\varepsilon(\phi) = \begin{cases} \frac{1}{2}[1 + cos(\pi\phi/\varepsilon)], & |\phi| \leq \varepsilon \\ 0, & |\phi| > \varepsilon \end{cases}, \tag{6}$$

and

$$H_\varepsilon(\phi) = \begin{cases} \frac{1}{2}[1 + \frac{\phi}{\varepsilon} + \frac{1}{\pi} sin(\pi\phi/\varepsilon)], & |\phi| \leq \varepsilon \\ 1, & \phi > \varepsilon \\ 0, & \phi < -\varepsilon \end{cases} \tag{7}$$

in which we set the parameter $\varepsilon$ to 0.5, so that the width of the approximation of $\delta$ is one pixel.

So given an initial LSF $\phi(x, 0) = \phi_0(x)$, the energy functional (1) can be minimized by solving the gradient flow $\frac{\partial\phi}{\partial t} = -\frac{\delta E_\varepsilon}{\delta\phi}$, which is of the form [1]

$$\frac{\partial\phi}{\partial t} = \delta_\varepsilon(\phi)\left[\lambda\nabla(g\mathbf{n}) + \alpha g + \beta\kappa\right] + \mu\nabla\left[p\prime\mathbf{n}\right]. \tag{8}$$

## 2 Parameters and initialisation

In Equation (8), we fix the parameter of the image term ($\lambda = 10$), and all the other weight parameters are determined relative to this one. In particular, the regularisation parameter $\mu$ is chosen fixed, $\mu = 0.001$ worked suitably for all our 3D tests. The most important parameters are thus the remaining weight parameters in the energy functional, $\alpha$ for the accelerating term and $\beta$ for the smoothing term. Different choices of these parameters lead to (slightly) different solutions, their choice is made by the averted user, depending on the specific question and situation.

In Section 2.1 we already illustrated the effect of these parameters on the solution of the level set method used to detect the tissue surface. Here we study the sensitivity with respect to the initialisation process.

First we created an artificial test image stack by taking mirror symmetries in a real tissue image (Supplementary Figure 1A), so that the object in the image doesn't touches the boundary of the image. We considered five different initialisations for the LSF, all computed as thresholds of the input image stack at different values, so that the initial contour is in the exterior of the tissue. Then we followed the evolution of the contours for the three representative parameter sets ($\alpha$,$\beta$) already presented in Section 2.1 (Supplementary Figure 1B-D).

Our results are briefly summarised in Supplementary Figure 2E. Given a parameter set ($\alpha$,$\beta$), notice the convergence to the same tissue volume regardless the initialisation. Furthermore, notice the acceleration property of $\alpha = 1$: when the accelerating term is turned on, the evolution is faster and the final object volume is lower. Notice also the effect of the smoothing term $\beta = 1$: the contour is smoothed out, the evolution is decelerated at the beginning, but the final tissue volume is still lower then the case when this term is turned off.

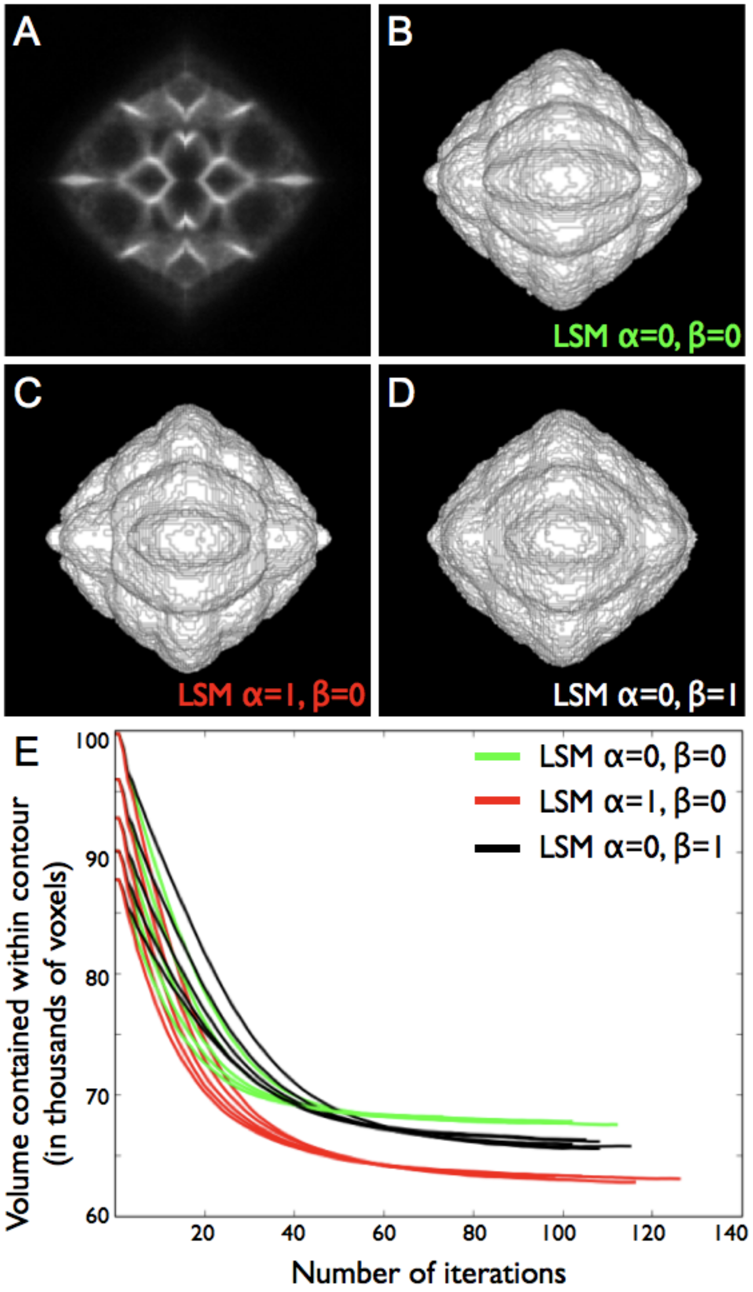


**Figure 1** **Parameter effects and sensitivity to initialisation. A.** Section of an artificial test image stack. **B-D.** Tissue contour detected by the level set method with different parameter sets. B:($\alpha = 0$,$\beta = 0$) - reference solution; C:($\alpha = 1$,$\beta = 0$) - the contour pushes into the valleys and the volume is lower as in B; D:($\alpha = 0$,$\beta = 1$) - the contour is smoothed out with respect to B; **E.** Sensitivity to initialisation. Curves present the evolution of the contour for five different initialisation thresholds in each of the three parameter sets shown in B-D.

**Author details**

**References**

1. Li, C., Xu, C., Gui, C., Fox, M.D.: Distance regularized level set evolution and its application to image segmentation. IEEE Trans. Image Process. **19**(12), 3243–3254 (2010)
